# The Molecular Weight Dependence of Thermoelectric Properties of Poly (3-Hexylthiophene)

**DOI:** 10.3390/ma13061404

**Published:** 2020-03-19

**Authors:** Saeed Mardi, Marialilia Pea, Andrea Notargiacomo, Narges Yaghoobi Nia, Aldo Di Carlo, Andrea Reale

**Affiliations:** 1 Department of Electronic Engineering, CHOSE—Centre for Hybrid and Organic Solar Energy, University of Rome Tor Vergata, via del Politecnico 1, 00133 Rome, Italy; saeed.mardi@gmail.com (S.M.); narges.yaghoobi.nia@uniroma2.it (N.Y.N.); aldo.dicarlo@uniroma2.it (A.D.C.); 2 Institute for Photonics and Nanotechnologies, CNR, 00156 Rome, Italy; marialilia.pea@ifn.cnr.it (M.P.); andrea.notargiacomo@ifn.cnr.it (A.N.)

**Keywords:** thermoelectrics, organic materials, poly (3-hexylthiophene) (P3HT), polymer chain, molecular weight

## Abstract

Organic materials have been found to be promising candidates for low-temperature thermoelectric applications. In particular, poly (3-hexylthiophene) (P3HT) has been attracting great interest due to its desirable intrinsic properties, such as excellent solution processability, chemical and thermal stability, and high field-effect mobility. However, its poor electrical conductivity has limited its application as a thermoelectric material. It is therefore important to improve the electrical conductivity of P3HT layers. In this work, we studied how molecular weight (MW) influences the thermoelectric properties of P3HT films. The films were doped with lithium bis(trifluoromethane sulfonyl) imide salt (LiTFSI) and 4-tert butylpyridine (TBP). Various P3HT layers with different MWs ranging from 21 to 94 kDa were investigated. UV–Vis spectroscopy and atomic force microscopy (AFM) analysis were performed to investigate the morphology and structure features of thin films with different MWs. The electrical conductivity initially increased when the MW increased and then decreased at the highest MW, whereas the Seebeck coefficient had a trend of reducing as the MW grew. The maximum thermoelectric power factor (1.87 μW/mK^2^) was obtained for MW of 77 kDa at 333 K. At this temperature, the electrical conductivity and Seebeck coefficient of this MW were 65.5 S/m and 169 μV/K, respectively.

## 1. Introduction

Conducting polymers have become promising materials for a wide range of applications, including thermoelectric generators, actuators, and supercapacitors. Novel features, such as their flexibility, light weight, nontoxicity, low thermal conductivity, and high chemical stability, make them promising candidates for a new generation of near-room-temperature thermoelectric materials [1]. Even though organic materials can easily decompose at high temperatures, most waste heat occurs at temperatures below 150 °C [2]. Among inorganic thermoelectric materials, Bi_2_Te_3_-based materials possess high thermoelectric properties at room temperature [3,4]. However, they have several drawbacks, such as scarcity of materials, high cost of production, toxicity, and processing difficulties [5], especially if low processing temperatures are considered, such as in the case of flexible plastic substrates. Organic thermoelectric materials can be used to harvest waste heat from the human body. Therefore, they can support low-power wearable devices, such as biosensors, watches, and toys [6]. Moreover, they are compatible with applications in the Internet of Things, since for these applications, large power generation is not required, but being lightweight and flexible are paramount [7,8,9]. The performance of thermoelectric materials depends on the dimensionless thermoelectric figure of merit (ZT), which is defined as ZT = S^2^σT/k, where σ (S/m), k (W/mK), S (V/K), and T (K) are the electrical conductivity, the thermal conductivity, the Seebeck coefficient, and the absolute temperature, respectively. The numerator of the figure of merit is called the power factor, which is proportional to the output power of the thermoelectric device.

In general, organic semiconductors have a low thermal conductivity even at highly doped levels, i.e., k < 1 W/mK [10]. Hence, the power factor S^2^σ is a good approximation for comparing organic thermoelectric materials. The poor electrical-transport properties of organic materials, including low electrical conductivity and low Seebeck coefficient, have prevented them from demonstrating improvement in their thermoelectric properties. The inverse interdependence between electrical conductivity and Seebeck coefficient limits improvement of the thermoelectric power factor [11,12]. Generally, increasing the charge concentrations usually leads to an increase in electrical conductivity; however, a corresponding decrease in the Seebeck coefficient is observed. In semiconductors, doping increases the charge concentrations and the Fermi level moves up (down) in energy towards the conduction (valence) band. In the case of a highly doped (degenerate semiconductor), the Fermi level moves into the conduction (valence) band, causing the density of electronic states above and below the Fermi level to come closer to being equivalent, so the Seebeck coefficient reduces [13]. However, the correlation between Seebeck coefficient and electrical conductivity could also be different. For example, Crispin et al. [14] reported that polymers can also be semimetallic, without having an energy bandgap, and a very low density of states at the Fermi level. They observed an enhancement of the Seebeck coefficient and electrical conductivity through molecular organization. The ZT of organic thermoelectric materials is low, usually two to three orders of magnitude lower than their inorganic counterparts [15]. However, in the past decade, great advances have been made in developing high-thermoelectric-performance organic materials. The highest ZT value among organic materials is 0.5, which was reported by Wang et al. in 2015 [16]. Moreover, various promising organic thermoelectric materials with remarkably high power factors (above 1000 μW/mK^2^) have been reported recently [17,18,19]. Apart from improving the thermoelectric properties of pristine polymers, another popular approach is the integration of polymers with nanofillers. In the composite, the σ and S could increase simultaneously, while the thermal conductivity remains nearly constant relative to the polymer without nanofiller [20]. Two different types of nanofillers are generally applied. The first type is carbon materials, such as carbon nanotubes [21], and graphene sheets [22]. The second one is inorganic materials, including tellurium nanowires [23], SnSe [24], and Bi_2_Te_3_ nanowires [25].

Conjugated polymers are of particular interest because of their excellent electronic properties, good physical and chemical stability, good solubility, processability, and the realization of low-cost and large-area products [26]. Through the simple tuning of their molecular structures, it is possible to modify their chemical and physical proprieties, so they offer great potential for meeting the requirements of various desired applications [27]. Moreover, carbon is one of the most abundant elements in nature; hence, the synthesis of organic electronic materials is more economical [27]. They have been widely considered for applications such as light-emitting diodes, thermoelectrics, transistors, bioelectronics, and solar cells. Poly (3-hexylthiophene) (P3HT) is one of the conducting conjugated polymers that has recently been thoroughly examined in various fields, including organic thermoelectric materials. P3HT shows several features that distinguish it from other conjugated polymers, such as high field-effect mobility, solubility in a variety of organic solvents, and chemical and thermal stability [28]. In planar polymers such as P3HT, generally, there are three efficient directions for charge transport: I) along the conjugated backbone, which has the highest charge transport due to the covalently linked conjugated units; II) along the π–π^*^ stacking axis, where the charge transport is slower; and III) along the lamellar stacking axis, where the slowest charge transport happens [29]. Therefore, the improvement in interchain order along the conjugated backbone could improve the charge transport. Chang et al. [30] investigated the correlation of interchain interaction and the field-effect mobility in the different molecular weights (MWs) of P3HT. Their results showed that upon increasing the MW, the interchain order improves and, as a result, the field-effect mobility increases. The effect of MWs of P3HT in organic and Perovskite solar cells has been considered by many authors [31,32]. Usually, increasing MW has a beneficial effect on solar cell performance. However, pristine P3HT has a low electrical conductivity, and it needs to be increased for many applications. For instance, in the use of P3HT as a hole transport layer in solar cells, the efficiency of the device shows remarkable enhancement using the two dopants, lithium bis(trifluoromethane sulfonyl) imide salt (LiTFSI) and 4-tert butylpyridine (TBP). According to the reported literature [33], LiTFSI increases the charge carriers, and the main role of TBP is to prevent the formation of individual isolated aggregates of LiTFSI, and to help the homogeneity of LiTFSI distribution [34,35]. TBP is therefore essential to have a homogeneous and interconnected polymer layer as well as ensuring the uniform electrical properties of the film. Moreover, from the electrical properties point of view, Guo et al. [36] found that TBP increases the mobility of the polymer, and their assumption was supported by X-ray diffraction (XRD) analysis and UV–Vis spectroscopy that indicated enhanced ordering of the P3HT polymer chains.

When considering the intrinsic thermoelectric properties of P3HT, it is commonly found in the literature that this material has a high Seebeck coefficient compared to other conducting polymers, such as PEDOT:PSS [37,38]. However, it has low electrical conductivity for thermoelectric applications, so it is important to increase electrical conductivity in P3HT layers. There have been many efforts made to address this problem, such as using different molecular configurations [39], varying the solvent [40], introducing fillers [20], or tuning the additive [41]. As mentioned before, the electrical properties of P3HT films with various MWs have been investigated in the literature. However, to the best of our knowledge, there is only one report which compares the thermoelectric properties of P3HT thick films with three different MWs [42]. Regardless of that report, the correlation between chain lengths, morphology, and thermoelectric parameters has not yet been completely studied. Herein, we deepen the effect of MW on the thermoelectric properties of P3HT thin films. P3HT films with various MWs ranging from 21 to 94 kDa were chosen. The electrical conductivity of pristine P3HT has been improved by adding LiTFSI and TBP. We found out that by increasing the MW, the electrical conductivity initially increases, then there is an inflection point, after which it decreases. However, the Seebeck coefficient has a monotonic decreasing trend as MW grows. Therefore, the highest power factor is obtained for MW of 77kDa. UV–Vis spectroscopy and atomic force microscopy (AFM) analysis were also performed to investigate the effect of MW on the structure and morphology of polymer films, respectively. Our results highlight the importance of MW tuning in high-performance organic thermoelectric materials.

## 2. Materials and Methods

Pristine P3HT (purchased from Merck (Darmstadt, Germany) in four different MWs of 21, 44, 77, and 94 kDa) was dissolved in 1:1 chlorobenzene/dichlorobenzene solvent mixture (10 mg/mL). Tert-butylpyridine (TBP) and lithium bis(trifluoromethane sulfonyl) imide salt (LiTFSI) solutions (520 mg in 1 mL of acetonitrile) were used as additives. The amounts of dopant for 1 mL P3HT solution were 91.2 and 96 µL for TBP and LiTFSI, respectively. The additives were added to the prepared P3HT solutions. P3HT solutions were stirred at 60 °C to improve the dispersion of the dopant. The samples were spin-coated on micro slides. For each MW, the spin-coating speed was tuned to achieve similar thicknesses for all the HTM layers. The films were then annealed at 150 °C for 10 min under an N_2_ environment. Silver electrical contacts (80 nm thick) for thermoelectric measurements were deposited onto the P3HT films via controlled thermal evaporation. A dedicated electrical system in a vacuum chamber was used to measure the electrical conductivity and Seebeck coefficients of thermoelectric devices. In-plane Seebeck coefficients and electrical conductivity were measured by placing the samples on two Peltier cells that forced cold and hot temperatures on the sample edges, via feedback control acting on the Peltier cells, with dedicated thermal probes (Pt100 thermistors) monitoring cold and hot temperatures of the samples. To improve heat conduction and temperature control between the samples and Peltier cells, a thermally conductive paste was used. The set-up was equipped with a Keithley 2420 source meter and Newport 8000 temperature controller, and all of these were operated with dedicated LabVIEW 2017 (National Instruments, Austin, TX, USA) software for feedback control of hot and cold sides of the samples. The electrical conductivities were calculated from the current-voltage measurement at different temperatures, based on the following equation:

$$\sigma =\frac{l}{R\;d\;t}$$

where *R*, *l*, *d*, and *t* represent the resistance (the slope of current-voltage curve), the distance between the two silver electrodes, the width of the silver electrode, and the thickness of the sample, respectively. Due to the high electrical resistivity of undoped P3HT, which is out of the measurement range of the source meter, the electrical conductivity and Seebeck coefficient of pristine P3HT have not been reported. The thickness of all samples was measured with a profilometer (Veeco-Digital Instruments (Munich, Germany) Dektak 150). The surface morphologies of the deposited films were measured by atomic force microscopy (AFM) using a Veeco-Digital Instruments (Munich, Germany) D3100 microscope equipped with a Nanoscope IIIa controller, in air environment, and employing tapping-mode probes with a nominal tip curvature radius of 5 ÷ 10 nm and spring constant ~40 N/m. The absorbance spectra were measured with an UV-Vis spectrophotometer (Shimadzu scientific instruments (Kyoto, Japan) UV-2550).

## 3. Results and Discussion

### 3.1. AFM Analysis and UV–Vis Spectroscopy

**Figure 1:**
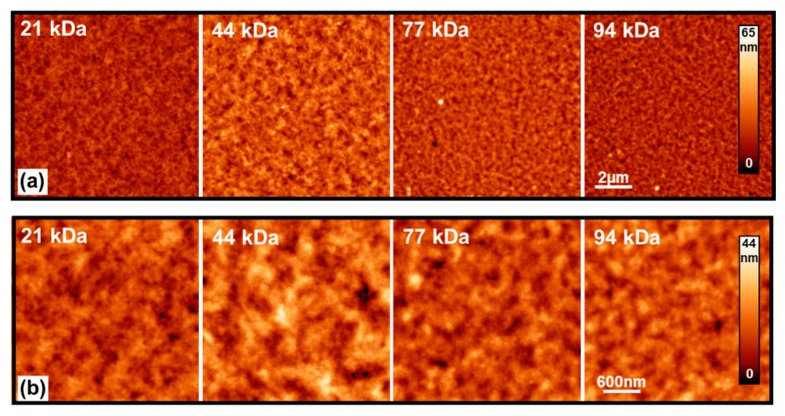
Atomic force microscopy (AFM) images of pristine poly (3-hexylthiophene) (P3HT) films deposited in different molecular weights (MWs). Panels (**a**) and (**b**) report AFM data with scan size of 10 and 3 μm, respectively. The scale-bar and color-bar are intended to apply for all the images in the respective panel.

Figure 1a,b shows the 2D AFM images of the surface topography of the pristine P3HT layers deposited on glass collected at a scan-size of 10 and 3 μm, respectively. The topographies exhibit no apparent MW-dependent behavior. However, the surface topography at higher MWs seems to become slightly smoother, which could be attributed to the increase of polymer chain lengths. It is also consistent with previously published reports describing the effect of MW on the electrical properties of P3HT films [42,43,44]. Variations in the topography among samples could be attributed to the difference in the evaporation rate of the solvent and the solidification of the polymer in different solutions. Indeed, the solutions of low-MW materials have lower viscosity and are more mobile compared to higher-MW samples.

**Figure 2:**
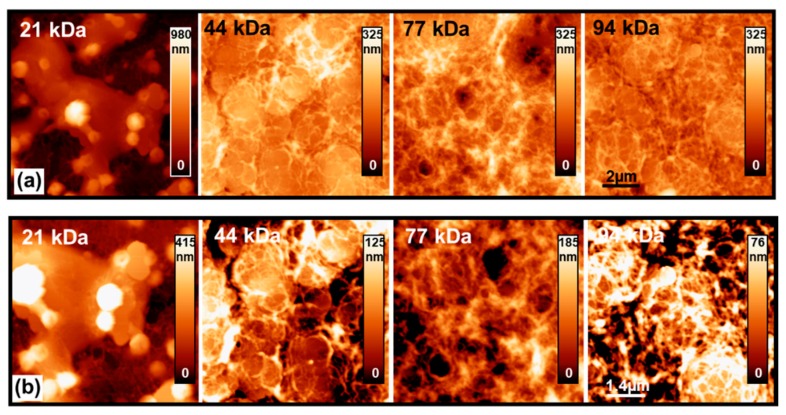
AFM images of doped P3HT films deposited in different MWs. Panels (**a**) and (**b**) report AFM data with scan sizes of 10 and 7 μm, respectively. In panel (**a**), 44 to 94kDa samples are shown with the same z-scale for a better comparison; in panel (**b**), the AFM data are reported with a properly fitted z-scale to highlight the details.

Figure 2a,b shows the AFM images of the doped P3HT (P3HT/Li + TBP) samples at 10 and 7 μm scan-size, respectively. In Figure 2b, the AFM data reported with a properly fitted z-scale for each single image highlighted the details of the P3HT morphological structures. The P3HT/Li + TBP films show quite a different morphology when compared to the pristine P3HT films. Moreover, the morphology of the films deposited at different MWs differs from sample to sample. The 21kDa sample shows apparent large and thick aggregates with superimposed globular structures which are uniformly distributed over the sample surface. As for the samples with MW in the 44–94 kDa range, the film topography is smoother, i.e., the thickness of the film is more uniform, and compact disc-like flat-top aggregates are present. A possible explanation for this phenomenon is that in the lowest MWs, which had shorter chain lengths, the additives were more likely to agglomerate, while in the higher-MW samples, the additives were distributed more homogeneously due to the longer chain lengths. These aggregates are covered by (or within) a polymeric network, which are more easily recognizable for the 44 and 94 kDa samples. Aggregates like those found in the samples are commonly observed by adding LiTFSI solved in acetonitrile [34]. The aggregation and morphology changes are typically associated with noncovalent interactions such as hydrogen bonds, electrostatic interactions, aromatic interactions, and hydrophilic/hydrophobic effects [45]. The large grainy aggregates might be attributed to the formation of solid LiTFSI within the P3HT layer, because acetonitrile has a lower boiling point compared to the other solvents [34,35,46]. As shown in Figure 2b, in all the samples, the P3HT polymeric network is clearly visible; it covers most of the sample surface aside from the large aggregates and in different amounts is present on top of the aggregates themselves. In particular, in the 44 and 94 kDa samples, distinguished single polymeric filaments are present on the flat surfaces of the aggregates, indicating of a low surface coverage. In contrast, in the 77 kDa sample, the aggregates are not apparent, and the polymeric film seems to be more uniform and cross-linked. These findings point to a better dispersion of the additives in the 77 kDa sample, indicating a lower aggregation and the formation of a more densely-packed polymeric network that may conceal small-size aggregates.

**Figure 3:**
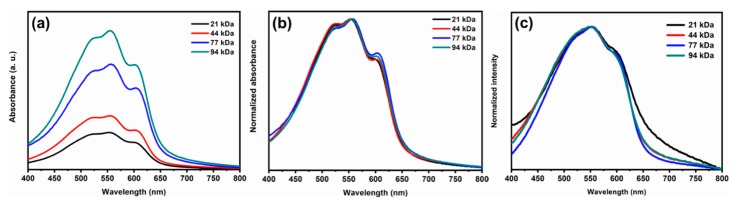
(**a**) Absolute and (**b**) normalized UV–Vis absorbance spectra of pristine P3HT in different MWs; and (**c**) normalized UV–Vis spectra of P3HT/Li + TBP in different MWs.

Figure 3 shows the UV–Vis absorbance spectra of the pristine P3HT and P3HT/Li + TBP layers. The thicknesses of the different P3HT layers with and without additives were measured to be about 40 ± 10 and 100 ± 10 nm, respectively. Generally, increasing the MW of P3HT results in an enhancement of the P3HT absorbance (Figure 3a) [31,47]. Normalized absorbance of the pristine P3HT layers is shown in Figure 3b. The featured peaks at ~607, ~558, and ~525 nm represent the typical behavior related to π–π* absorption transitions for all MWs. The peaks at 525 and 607 nm originate from the optical interband transition from the π to π* orbitals, and the strong interchain interactions. Therefore, the absorbance peaks at 525 and 607 nm provide information on the degree of conjugation of the P3HT chains and the degree of interchain order [31]. The absorbance peak at 607 nm increased with increasing MWs up to 77 kDa. A further increase in MW reduces the intensity of this peak. This shows that the highest level of interchain order happens in the 77 kDa sample. Figure 3c shows UV–Vis spectra of P3HT/Li + TBP layers as a function of MW. Unlike the pristine P3HT layers, they have a similar intensity at 525 and 607 nm. Due to the presence of additives and aggregates, the polymer chains are unpacked and inhomogeneous. This should impact the optical transitions, so the featured peaks of the P3HT layers might be smeared out.

### 3.2. Electrical Transport Properties

**Figure 4:**
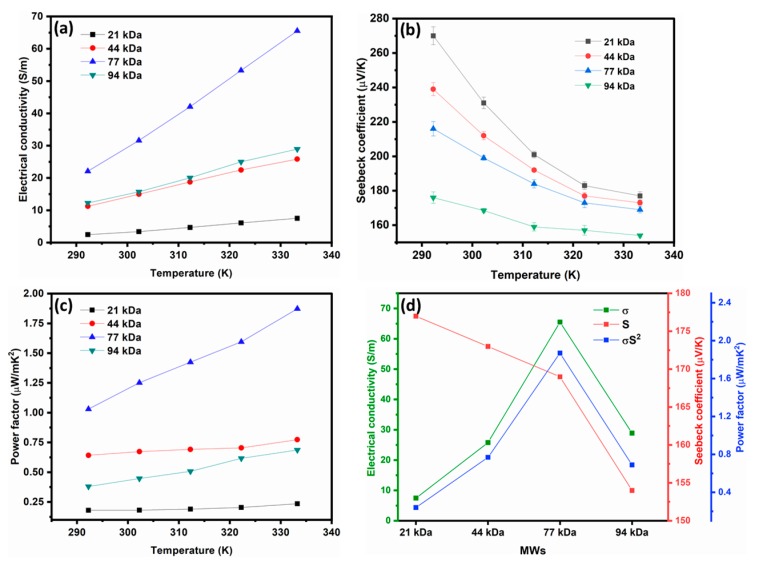
(**a**) Electrical conductivity, (**b**) Seebeck coefficient, (**c**) and power factor versus temperature for P3HT/Li + TBP films with different MWs, and (**d**) the comparison of thermoelectric properties of samples with different MWs at 333 K. Error bars indicate the standard deviation of three experimental replicates.

The Seebeck coefficient, electrical conductivity, and power factor as a function of temperature for different P3HT layers are displayed in Figure 4. As shown in Figure 4a, the electrical conductivity increases with an increase in temperature. Comparing the electrical conductivity between the different MWs shows that increasing the MW increases the electrical conductivity up to 77 kDa, and then it decreases at 94 kDa. The electrical conductivity behavior is consistent with the discussion on the chain length of P3HT, i.e., a higher chain length facilitates the charge transport. As the MW grows, the chain length increases and the gaps between them are reduced. This provides a larger ordered region in the polymer to facilitate charge transport. However, we note that the 94 kDa sample presents lower electrical conductivity compared to the 77 kDa one. Thus, we can argue that there is an optimal value of the MW and of the chain length for polymer performance. Indeed, excessive increases in MW may cause folding of the polymer chain and decrease of the ordering level of the polymer. Moreover, this could possibly lead to the formation of amorphous domains, consequently lowering the amount of hopping between charge carriers. This is also consistent with the observations on the AFM data shown in Figure 2, i.e., in the higher-MW samples, the additives distributed more homogeneously. The slope of the Seebeck coefficient versus temperature decreases with an increase in temperature, as shown in Figure 4b. This behavior was observed in some conducting polymers, and it can be attributed to hopping conductivity via the nearest neighbors [48]. Figure 4b demonstrates that the Seebeck coefficient continues to reduce as MW increases.

The reduction of Seebeck coefficients was expected as higher MWs led to enhanced electrical conductivity and there is usually a trade-off between electrical conductivity and Seebeck coefficient. However, the 94 kDa sample shows lower values of electrical conductivity and Seebeck coefficient compared to the 77 kDa sample. This might be attributed to the decrease in the interchain order and the formation of amorphous areas [40]. The reduction rate of the Seebeck coefficient is slower than the growth rate in electrical conductivity. Therefore, in our experiment, the dependency of power factor vs. MW is mainly determined by electrical conductivity, and hence, the maximum thermoelectric power factor reaches 1.87 μW/mK^2^ at 333 K for the 77 kDa sample, as shown in Figure 4c. The comparison of all parameters at 333 K is shown in Figure 4d. At the given temperature, the power factor reaches a maximum of 77 kDA and then drops again.

## 4. Conclusions

In summary, we investigated the relationship between the thermoelectric parameters and the molecular weight (MW) of P3HT thin films. The electrical properties of P3HT were improved by LiTFSI and TBP as additives. We showed the relevant effects of MW on the structure of polymer films, such as morphological compactness, distribution of additives, and the degree of interchain order. The results of AFM analysis clearly illustrated that the MW had an effect on the distribution of additives, which are distributed more homogeneously in high-MW samples. The comparison of AFM images of doped samples revealed that in the 77 kDa sample, the polymeric network has a more uniform and densely-packed structure and points to a lower aggregation of the additives.

UV–Vis spectroscopy also confirmed that the degree of interchain order has a maximum of around 77 kDa and then decreases for the highest MW. Moreover, a clear correlation between MW and thermoelectric properties was demonstrated, showing that there is a trade-off between MW and thermoelectric properties in P3HT. The Seebeck coefficient had a declining trend as the MW grew. However, the highest electrical conductivity occurred for 77 kDa, so a MW around 77 kDa was found to be optimal with respect to the power factor. This research emphasizes the importance of considering MW as a key parameter for optimization of the thermoelectric performance of P3HT.
